# Geographic Distribution, Age Pattern and Sites of Lesions in a Cohort of Buruli Ulcer Patients from the Mapé Basin of Cameroon

**DOI:** 10.1371/journal.pntd.0002252

**Published:** 2013-06-13

**Authors:** Martin W. Bratschi, Miriam Bolz, Jacques C. Minyem, Leticia Grize, Fidèle G. Wantong, Sarah Kerber, Earnest Njih Tabah, Marie-Thérèse Ruf, Ferdinand Mou, Djeunga Noumen, Alphonse Um Boock, Gerd Pluschke

**Affiliations:** 1 Swiss Tropical and Public Health Institute, Basel, Switzerland; 2 University of Basel, Basel, Switzerland; 3 FAIRMED Africa Regional Office, Yaoundé, Cameroon; 4 Bankim District Hospital, Bankim, Cameroon; 5 National Committee for Leprosy and Buruli Ulcer Control, Department of Disease Control, Ministry of Public Health, Yaoundé, Cameroon; University of Tennessee, United States of America

## Abstract

Buruli ulcer (BU), a neglected tropical disease of the skin, caused by *Mycobacterium ulcerans*, occurs most frequently in children in West Africa. Risk factors for BU include proximity to slow flowing water, poor wound care and not wearing protective clothing. Man-made alterations of the environment have been suggested to lead to increased BU incidence. *M. ulcerans* DNA has been detected in the environment, water bugs and recently also in mosquitoes. Despite these findings, the mode of transmission of BU remains poorly understood and both transmission by insects or direct inoculation from contaminated environment have been suggested. Here, we investigated the BU epidemiology in the Mapé basin of Cameroon where the damming of the Mapé River since 1988 is believed to have increased the incidence of BU. Through a house-by-house survey in spring 2010, which also examined the local population for leprosy and yaws, and continued surveillance thereafter, we identified, till June 2012, altogether 88 RT-PCR positive cases of BU. We found that the age adjusted cumulative incidence of BU was highest in young teenagers and in individuals above the age of 50 and that very young children (<5) were underrepresented among cases. BU lesions clustered around the ankles and at the back of the elbows. This pattern neither matches any of the published mosquito biting site patterns, nor the published distribution of small skin injuries in children, where lesions on the knees are much more frequent. The option of multiple modes of transmission should thus be considered. Analyzing the geographic distribution of cases in the Mapé Dam area revealed a closer association with the Mbam River than with the artificial lake.

## Author Summary

Buruli ulcer (BU) is an infectious disease caused by *Mycobacterium ulcerans* that is affecting mostly children in endemic areas of West Africa. Proximity to slow flowing water is a risk factor, but the exact mode of transmission of BU remains unclear. Man-made environmental changes, such as sand mining, damming of rivers and irrigation have been implicated with increases in disease incidence. Here, we report findings from a survey for BU and continued case detection thereafter in the Bankim Health District of Cameroon. In this area, the local population believed that the damming of the Mapé River has led to the emergence of BU. In 28 months we identified 88 laboratory confirmed cases of BU. Studying these cases, we found that the age adjusted cumulative incidence of BU in the elderly is similar to that in children and that the distribution pattern of BU lesions neither matches mosquito biting patterns nor the distribution of small skin injuries. Multiple modes of transmission should therefore be considered. Our data further showed that the patients appear to have closer contact to the local Mbam River than to the artificial Mapé dam reservoir.

## Introduction

Buruli ulcer (BU), a neglected tropical disease (NTD) of the skin, is caused by *Mycobacterium ulcerans* [1] and if untreated, can lead to disability. Worldwide, local BU incidence rates are highest in West Africa and Australia, where the classical lineage of *M. ulcerans* is found [2]–[4] and the disease occurs at different foci in the endemic countries. Both sexes can be affected by the disease and although individuals of all ages can get BU, most of the patients are less then 15 years old [5]. In Cameroon, BU was first described in 1969 in the Nyong river valley where during a cross-sectional survey in 2001, a total of 436 clinically diagnosed cases of active or inactive BU were found [6]. Since then, the Bankim Health District (HD) has been identified as an additional BU endemic area in Cameroon [7]. In this area, where our research has been carried out, the local population suspects that the creation of an artificial lake, by damming of the Mapé River in 1988, has led to an increase in BU incidence. Risk factors for BU include proximity to slow flowing water, poor wound care and not wearing protective clothing [8]. However, the exact mode of transmission has not yet been elucidated [9], [10]. Clinically, BU presents with symptoms ranging from nodules, plaques and oedemas to ulcers [11]. The cytotoxic and immunosuppressive toxin, mycolactone, uniquely produced by *M. ulcerans*, is believed to account for most of the pathology of BU [12]. The severity of cases is classified into three categories, with ‘1’ being patients with small (≤5 cm dimeter) lesions, ‘2’ patients with medium size lesions (5–15 cm) and ‘3’ being patients with large (>15 cm) lesions, multiple lesions or lesions at critical sites [13]. Many BU cases identified in rural areas are still diagnosed based on clinical symptoms only, although the use of laboratory diagnosis is highly recommended by the World Health Organization (WHO). In 2004, the WHO introduced the use of the combination of streptomycin and rifampicin given daily for 8 weeks as treatment [14]. However, surgery and wound management remain critical aspects of BU care [15], [16].

During our investigations of BU in the Bankim HD, we also examined the local population for two other NTDs of the skin, namely yaws and leprosy. Yaws is caused by *Treponema pallidum* (*T. pallidum*) subspecies *pertunu*, and is transmitted through skin and mucous membrane contact [17], [18]. After an initial single lesion, the disease progresses to secondary multiple lesions and in about 10% of cases it causes permanent disability [18]. Leprosy is caused by *Mycobacterium leprae*, which is believed to be transmitted by the respiratory route and can cause major disabilities through nerve damage. Diagnosis of yaws and leprosy relies mainly on physical examinations [17], [19] and treatment of both diseases is feasible with antibiotics [17], [20].

The objectives of the present study were i) to conduct an exhaustive survey for BU, yaws and leprosy in the Bankim HD; ii) to continuously monitor the occurrence of BU in the Mapé Dam area; and iii) to examine the age distribution, geographic origin and distribution of lesions of the real-time polymerase chain reaction (RT-PCR) confirmed cases of BU to underpin future environmental and social science studies.

## Materials and Methods

### Ethical statement

Approval for the survey and the subsequent continuous enrolment of cases was obtained from the Cameroon National Ethics Committee (N°041/CNE/DNM/09 and N°172/CNE/SE/2011) and the Ethics Committee of Basel (EKBB, reference no. 53/11). Participation in all aspects of the study was voluntary and all patients, independent of their study participation, were treated according to national treatment standards. All clinically confirmed cases who participated in the study provided written informed consent.

### Study area

**Figure 1:**
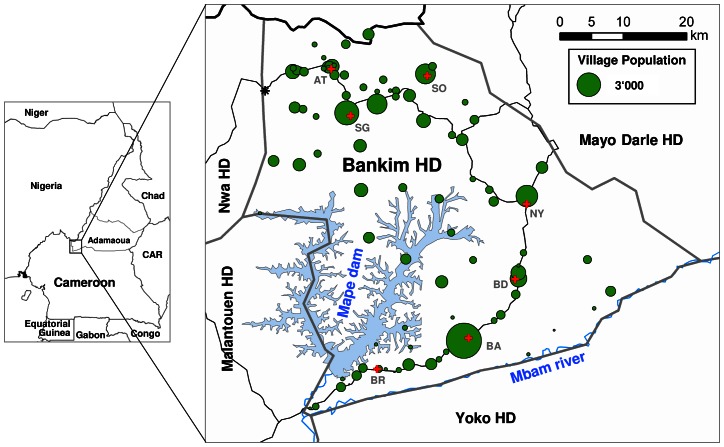
Environmental features and population of the Bankim HD. The Bankim HD is located in the South-Western corner of the Adamaoua Region of Cameroon and encompasses most of the Mapé basin. The main environmental features of the area are the Mapé Dam reservoir and the Mbam River. The Bankim HD consists of 7 HA (red crosses depict the location of the main HC in each of the HA; BR: Bankim Rural; BA: Bankim Urban; BD: Bandam; NY: Nyamboya; SO: Somié; SG: Songkolong; AT: Atta) and is surrounded by four other HD. In early 2010 we conducted an exhaustive house-by-house survey and examined a total of 48'962 individuals in all HA of the district; population sizes of villages based on this survey are indicated by green circles. The village of Koumtchoum (black star; estimated population of 550) as a whole refused to participate in the survey and the village of Djaouro Tchi Arouna (470 inhabitants, located in the Somié HA between the HC of Somié and the town at the Nigerian border to the North-West of it) could not be mapped.

The study was conducted in the Mapé Dam region of Cameroon (Figure 1) at two different geographical scales. The initial phase of the study was conducted in the Bankim HD which consists of seven Health Areas (HA): Atta; Songkolong, Somié, Nyamboya, Bandam, Bankim Urban and Bankim Rural. The health care infrastructure of the Bankim HD consists of one public district hospital, six primary and four private health centres (HC). All of these facilities employ two medical doctors and approximately 30 nurses. For the later part of the study, bordering regions in the 4 HD surrounding the Bankim HD (Nwa HD, Malantouen HD, Mayo Darle HD, Yoke HD) were also included in the study area. The main environmental features of the area are the Mapé Dam and the Mbam River.

### Survey procedures

In early 2010 (March 22 to April 19), we conducted an exhaustive cross-sectional house-by-house survey for BU, leprosy and yaws in the 88 villages of the Bankim HD (Figure 1). Eleven teams of three trained field workers, namely one local nurse and two local community relays, were employed to interview all inhabitants. Field workers were trained for two days on the use of the questionnaire and the clinical signs of the three diseases investigated. At the household level, demographic information of all inhabitants was collected and posters with photographs of the clinical presentations of the diseases were shown. Households with suspected cases were re-visited by staff with extensive experience in the diagnosis of BU, leprosy and yaws. From clinically confirmed BU cases, samples were collected for laboratory confirmation as follows. Two or three dry swabs were collected from ulcerative lesions or a fine needle aspirate (FNA) was drawn from non-ulcerative lesions [21]. To facilitate handling of FNA samples, they were transferred onto a swab.

### Prospective BU surveillance

Following the survey, we continued to monitor the occurrence of all new cases of BU in the Bankim HD by community and HC based case referral and regular supervision until the end of June 2012. For this, a health worker, trained and experienced in the diagnosis of BU, regularly visited all HC in the Bankim HD and areas of the adjacent Malantouen HD. During these visits, suspected cases who independently came forward or who were referred to the HC by community or family members, were evaluated and if clinically confirmed, asked to come for treatment. Before treatment, swabs or an FNA were collected for laboratory confirmation as described above. In addition to demographic and clinical information, the houses where the patients lived for at least a year before disease onset were mapped using a GPS device. From the GPS device, coordinates were only recorded once the GPS receiver showed an accuracy of below 10 m. Details of the location of the lesions on the patient's bodies were also collected and documented by photographs. Both clinically confirmed BU cases identified in the survey and during the continuous case detection were included in the cohort of patients investigated here.

### Laboratory confirmation of BU cases

Samples were locally stored at 4°C before transport to the laboratory where definite BU diagnosis was obtained by insertion sequence (*IS*) 2404 RT-PCR. Analysis was done according to the protocol developed by Fyfe *et al.* [22], [23]. In brief, swabs were transferred into glass bottles containing glass beads with 2–5 mL of PBS, and the bottle vortexed for 1.5 minutes. From 1 mL of the solution, DNA was extracted and RT-PCR performed. DNA was amplified in a StepOnePlus Real-Time PCR System (Applied Biosystems) and data analyzed using the Applied Biosystems StepOne Software (2.2.2).

### Analysis of lesion distribution

Using published age specific relative body surface areas (RBSA) [24] and the number of patients in each of the age groups, the weighted average RBSAs of a model person (all ages), a model child (<15), and a model adult (≥15) were computed. If required to perform a Fisher's exact test, RBSA were converted to counts which add up to the observed number of lesions. The shape file used to analyze lesion localizations is found in Dataset S1.

### Statistical analysis

Continuous variables were summarized as means and standard deviation or medians and interquartile ranges and categorical ones as counts and percentages. The Fisher's exact or Chi-squared tests were used to compare categorical characteristics between groups and Student t-tests or Mann-Whitney U-test in the case of continuous variables. Multiple comparisons were adjusted for using a Bonferroni correction. The software, SAS (SAS Institute, Cary, USA; release 9.3), RStudio (RStudio, Boston, USA, version 0.95.262) and R (The R Foundation for Statistical Computing; version 2.15.1) were used to perform the statistical analysis. Geographic data and the localisation of lesions were analyzed with ArcGIS ArcMap (Economic and Social Research Institute, Redlands, USA; version 10.0).

## Results

### Survey for BU, leprosy and yaws in the Bankim HD

In the course of the survey, a total of 48 962 individuals in 9 344 households (Figure 1) were interviewed. The population of one village (approximately 550 people) refused to participate in the study. Assessing demographics and living conditions in the Bankim HD (Table 1), we found that the local population is very young with an average age of 19.3±17.0 (median = 14.0, interquartile range = 6.0 to 28.0), that 51.4% of the population are women and that overall, 61.2% of the population have attended school at some point in their lives. We further observed that Christianity is the most common (64.9%) religion and that, apart from the young members of society which are either students (32.2%) or children (23.5%), the most common professions in the district are farming (16.9%) and household work (17.4%). In terms of living conditions we found that there are on average 5.2 individuals living in each household and 26.8% of the households have a mosquito net. Further, our data showed that only 38.3% of the population have access to clean drinking water that comes at least from a fortified well and that the roofs and floors of the local houses are often very poorly constructed. Table 1 also shows that, the main local differences in the level of development in the HD exist between the six rural HA and the Bankim Urban HA (BA HA), which includes the town of Bankim (77.4% of the BA HA population) and nine small settlements around it. The higher level of development in the BA HA is reflected by the significantly higher percentage of people having gone to school at some point in their lives (p-value<0.0001) or by the significantly better access to clean drinking water (p-value<0.0001). Furthermore, in the BA HA the proportion of houses with better flooring (p-value<0.0001) and walls (p-value = 0.0037) is also significantly higher compared to the other HA.

**Table 1:** Sociodemographic characteristics of the Bankim Health District population.

	Health Areas							
Characteristic	AT	SG	SO	NY	BD	BA	BR	Total
**Individuals**								
Inhabitants surveyed (n)	7 621	11 346	7 308	5 602	5 421	8 514	3 150	48 962
Age in years (mean ± SD)	19.9±17.0	19.8±17.2	20.2±18.2	18.2±16.3	17.4±16.5	19.3±16.1	19.1±16.5	19.3±17.0
Gender (% female)	49.8	50.9	52.9	53.1	51.7	51.1	51.1	51.4
Education* (%)	62.2	64.9	55.9	53.8	49.8	72.0	61.5	61.2
Religion (%)								
Christian	72.9	65.2	61.2	59.6	52.2	70.6	69.6	64.9
Muslim	26.7	34.6	38.8	40.4	47.8	28.7	30.2	34.8
Profession (%)								
Farming	25.6	13.3	17.0	12.5	13.7	12.7	32.9	16.9
Fishing	0.1	2.4	0.1	2.2	1.1	0.7	2.2	1.2
Household work	13.4	21.3	21.9	18.9	21.6	13.3	4.1	17.4
Student	32.9	33.6	26.8	30.6	24.0	40.5	33.2	32.2
Child	21.3	22.4	27.5	26.1	32.6	16.2	23.0	23.5
**Households**								
Number of households (n)	1788	2100	1332	1053	942	1532	597	9344
Individuals per household (mean ± SD)	4.3±2.9	5.4±2.9	5.5±3.3	5.3±3.0	5.8±3.7	5.6±3.4	5.3±2.9	5.3±3.2
Mosquito net** (%)	16.1	22.3	28.6	36.9	31.6	21.8	57.6	26.8
Clean drinking water*** (%)	27.0	34.5	47.9	17.2	25.0	76.7	23.2	38.3
Concrete or mood walls (%)	99.1	97.3	98.6	99.2	91.3	98.8	98.5	97.8
Tile or metal sheet roof (%)	40.6	40.8	32.0	39.4	31.4	78.1	42.8	44.7
Cemented or tailed floor (%)	8.0	10.9	7.8	9.7	6.8	41.6	11.6	14.3

* Attended school anytime during their life.

** Lived in household with a mosquito net.

*** Had access to drinking water from a tap or a concrete fortified well.

SD: standard deviation.

AT = Atta.

SG = Songkolong.

SO = Somié.

NY = Nyamboya.

BD = Bandam.

BA = Bankim Urban.

BR = Bankim Rural.

In the survey, we identified 32 cases of leprosy, 29 cases of yaws and 25 cases of BU based on clinical symptoms. With 32 cases of leprosy, the population-adjusted prevalence was at 6.5 cases per 10'000. The majority (70%) of the identified leprosy cases suffered from the multibacillary form of the disease and 22% of them were previously known but had abandoned their treatment and needed treatment re-initiation. Of the 29 yaws cases identified, 28% presented with the advanced symptom of hyperkeratosis and of the BU cases, 23% (6 cases) could be re-confirmed by RT-PCR.

### Laboratory confirmation of BU

**Figure 2:**
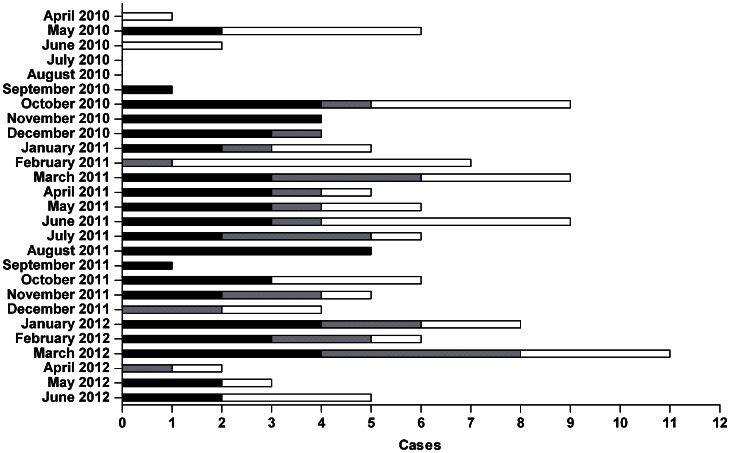
Identification of BU cases in the Mapé Basin following the survey. In the 27 months following the exhaustive survey all clinically diagnosed BU cases in the Bankim area were included in a cohort study. Cases are separated into the RT-PCR confirmed cases which occurred inside (black) and outside (grey) of the Bankim HD. RT-PCR negative and non-laboratory examined cases occurring anywhere in the Mapé basin during the same time period are also shown (white).

In the five months after the survey (April 2010 to August 2010), only two new RT-PCR reconfirmed BU cases were identified (Figure 2). Following this lag, between September 2010 and June 2012 (22 months) there was a steady flow of about 2.5 new RT-PCR confirmed BU cases per month from the Bankim HD. During this period, RT-PCR confirmed BU patients from the surrounding HDs (about 1.2 per month) also reported to BU treatment facilities in the Bankim HD. Overall, our study identified 157 clinically confirmed cases of BU of which 88 (56%) could be confirmed by RT-PCR. Of the non-confirmed patients, 48 (31%) tested negative in RT-PCR and of 21 patients (13%) no samples were collected. Gender ratio, age distribution, average disease duration prior to consultation, and proportion of category 3 cases were comparable between the RT-PCR positive and negative patients. Only age differed significantly (p-value = 0.034) between the RT-PCR confirmed and the non-confirmed cases with the average age of the confirmed cases being 21.2 and that of the non-confirmed ones being 29.3. To ensure the reliability of our conclusions we focused the remaining analysis only on the 88 RT-PCR confirmed BU cases. Age distribution (p-value = 0.4754) and professions (p-value = 0.5161) did not differ significantly between the population of the Bankim HD and the confirmed BU patients. The gender distribution among patients was moderately different (p-value = 0.061) from that of the overall population with a larger proportion of males among the confirmed BU cases.

### Spatial distribution of BU cases in the Mapé basin

**Figure 3:**
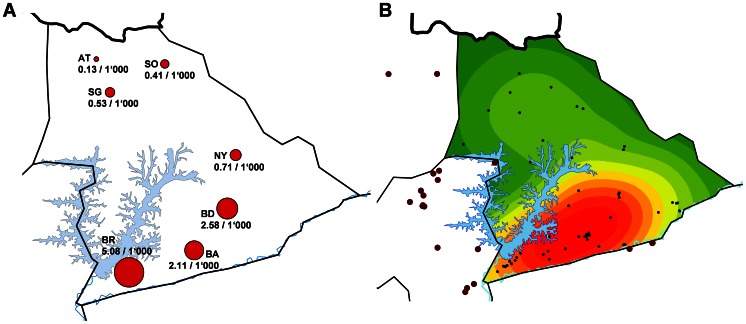
Geographic distribution of BU in the Mapé Basin. Based on the HA of origin of the 62 BU cases who originated within the Bankim HD and the population data collected in the survey, we computed the per HA cumulative incidence rate of BU in the Bankim HD (A). For detailed analysis, the places of residence of 58 (black points) of the 62 cases from the Bankim HD were mapped using a GPS device (B). For the remaining 4 RT-PCR confirmed cases which occurred inside the Bankim HD (3 from the Bandam HA, 1 from the Somié HA) we could not identify their home. Using a Kernel function the density of BU in the Bankim HD was computed based on the mapped cases (red: highest BU density). Panel B further shows the places of residence of 21 of the 26 RT-PCR confirmed cases of BU who originated from outside of the Bankim HD (brown points). For three of the remaining cases (1 from the Malantouen HD, and 2 of unknown origin) we could not identify their exact origin and two additional cases (1 each from the Nwa HD and Mayo Darle HD) are not shown because they originated from outside of the region shown on the map.

To better describe BU epidemiology in the Mapé basin we set out to identify the exact geographic origin of all 88 laboratory confirmed cases in our cohort. Based on information from the patients or their close relatives we were able to determine the HD of origin for 86 (98%) of the cases (Figure S1). For the remaining 2 cases we could only determine that they did not live in the Bankim HD for the year before the onset of symptoms, but we could not conclusively determine which HD they were from. Studying the distribution of cases by HD, we found that the proportion of category 1 cases among the patients originating from inside the Bankim HD (24/62) was significantly higher (p-value = 0.039) compared to the cases from the surrounding HDs (5/26; Figure S1). For the 62 cases that originated from within the Bankim HD we were also able to determine their HA of origin. Using the population data as collected by the survey, we were then able to calculate the cumulative incidence rate of BU per HA in the Bankim HD during our study. As shown in Figure 3A, the cumulative incidence rate of BU in the Bankim HD is highest in the BR HA (5.08/1'000). The cumulative incidence rate in this HA is significantly higher compared to all other HA in the HD (p-value<0.001). Interestingly the cumulative incidence rate is also significantly higher in the southern HA (BR, BA, BD, NY) compared to the northern HA (AT, SO, SG) of the Bankim HD (p-value<0.001) (Figure 3A). Finally, for more detailed spatial analysis, the exact domiciles of 79 (89.8%) of the confirmed BU cases were mapped (Figure 3B). For 7 of the remaining cases (Bankim HD: 3 from the Bandam HA, 1 from the Somié HA; surrounding HD: 1 from the Malantouen HD, and 2 of unknown origin) we could not conclusively identify the exact house where they lived before the onset of BU. An additional two cases (1 each from the Nwa HD and Mayo Darle HD) are not considered in the analysis because they originated from outside of the Mapé basin. Based on the known exact origin of the cases that came from within the Bankim HD (n = 58) and who were therefore identified by the same case finding strategy, a Kernel function was used to compute the density of BU in the Bankim HD (Figure 3B). This BU density map shows that most of the cases occur in the southern part of the Bankim HD, particularly along the Mbam River and in the area between the Mapé Dam reservoir and that river. The exact origins of 80.8% (21/26) of the BU cases from outside of the Bankim HD, indicate that the local BU focus expands outside of the Bankim HD, in particular westwards into the Malantouen HD (Figure 3B).

### Age and gender distribution of cases

The median age of the 88 RT-PCR confirmed cases was 12.5 (interquartile range = 8.0 to 30.0). The age of patients ranged from 0.5 to 73, 52 out of 88 (59.1%) were children (age <15) and 11 (12.5%) were older than 50. The gender ratio of all cases was 1.44 male/female. In children this ratio was 1.89, in the 15 to 50 year olds it was 0.79 and in the above 50 year olds, it was 1.75. The age dependent variation in the gender ratio was not statistically significant (p-value = 0.20).

**Figure 4:**
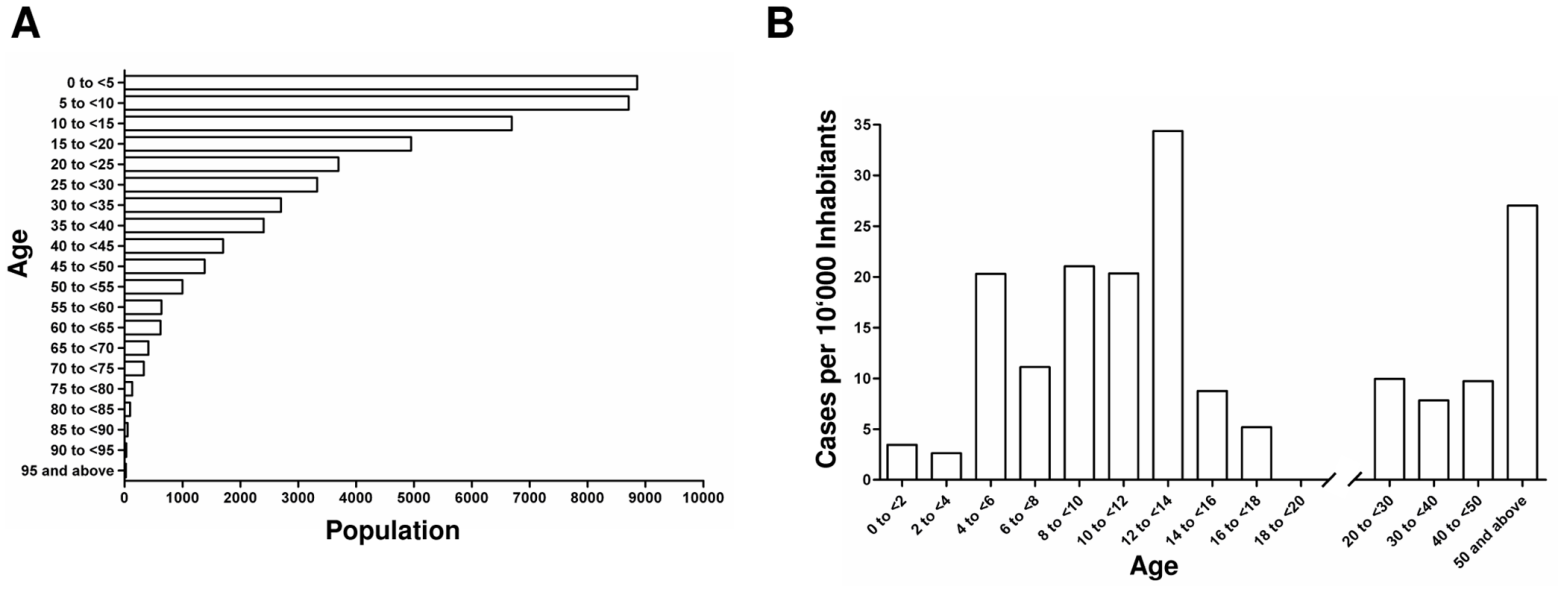
Population age distribution and age adjusted cumulative BU incidence rate in the Bankim HD. In the course of the exhaustive survey, data on the population age structure of the Bankim HD were collected (A). Using this age distribution and the ages of the RT-PCR confirmed BU cases which occurred inside of the Bankim HD (n = 62), the age adjusted cumulative BU incidence rate (cases per 1'000 inhabitants) for the duration of the study could be computed (B).

With the ages of the 62 (70.5%) cases of BU which originated from within the Bankim HD and the population age distribution as collected in the survey (Figure 4A and Table 1), we computed the age adjusted cumulative incidence rate of BU in the Bankim HD for the period of the study. As shown in Figure 4B, we observed a low age adjusted cumulative incidence rate of BU in individuals aged below four years. The rate then peaked in children aged between four and <14 years of age, with the 12 to <14 year olds particularly affected (34.4 cases per 10'000 inhabitants). Interestingly, the age adjusted cumulative incidence rate peaks again in the over 50 year olds (27.0 cases per 10'000 inhabitants; Figure 4B).

### Localisation of BU lesions

**Figure 5:**
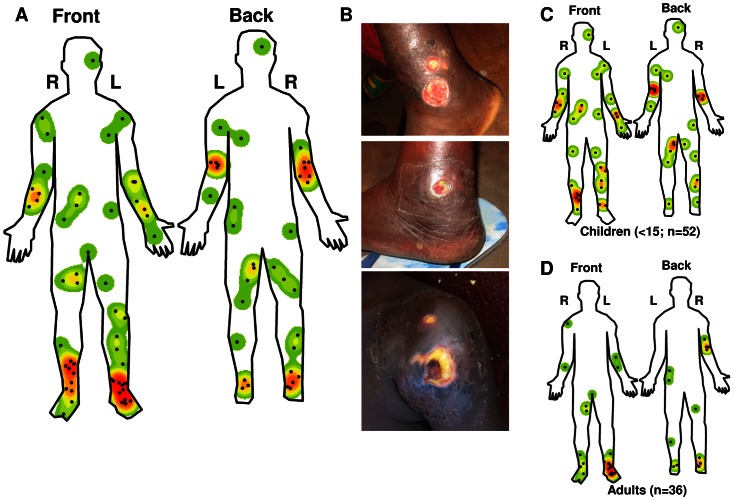
Lesion localization. The localization of the lesions of all the RT-PCR confirmed BU patients (88) were mapped in detail and Kernel function was used to create a heat map of the lesion distribution (A). The localization of the lesions on the front and back and left (L) and right (R) of the patient's bodies are shown. Studying the distribution of lesions, it was noted that they often occur at joints (B, example of two lesions on the ankle and one on the shoulder). Distribution of lesions in children (C, n = 52) and adults (D, n = 36) were also analyzed separately.

In the laboratory confirmed BU patients studied here, 49/88 (55.7%) lesions occurred on the lower limbs, 27/88 (30.7%) on the upper limbs, 2/88 (2.3%) on the head and neck and 10/88 (11.4%) on the trunk. One of the trunk lesions occurred on the genitals. Two patients had multiple lesions and only the initial lesion was considered for analysis. The distribution of lesions differed significantly (p-value<0.001) from the relative body surface area (RBSA; Table S1). Interestingly, most of the lesions (52.3%) occurred in close proximity to joints with clusters around the ankles (19.2%) and elbows (15.9%; Figure 5A, 5B and Table S2). When analyzing the occurrence of lesions on the different body parts, we did not observe any statistically significant difference between lesions occurring on the right or left or front or back of the patients. However, when analyzing the occurrence of lesions on the joints, we did observe a statistically significant difference between the lesions on the front or back of the joints (p-value = 0.012), in particular there was a significant difference between the occurrence of lesion on the front or back of the elbow (p-value = 0.005). No such difference was observed when analyzing the joint lesions on the right of left of the patients' bodies. Analyzing the distribution of the lesions by body part, we found a moderately significant difference between males and females (p-value = 0.076) with the percentage of lesions on the trunk being significantly higher (p-value = 0.033) in males (Table S1).

The distribution of lesions by body parts in children (Figure 5C and Table S1) was significantly different (p-value = 0.009) from the RBSA of children. Interestingly, only children (n = 2) had lesions on the head and neck. Overall, the lesions appear more dispersed in children (Figure 5C and 5D). While, the difference in the general lesion distribution by body parts between adults and children (Table S1) was not statistically significant (p-value = 0.154), there was a significant difference (p-value = 0.011) between the distribution of lesions at joints in children and adults. In particular, most joint lesions in adults occurred at the ankle (36.1%), whereas most joint lesions in children occurred at the elbow (19.2%). Finally in adults, lesions occurred mainly on the front and back of the feet and the distribution also differed significantly (p-value = 0.004) from what is expected based on the RBSA of adults (Figure 5C).

## Discussion

The 2011 Cameroon Demographic Health Survey (DHS) examined approximately 22'000 adults (>14 years old) and found that the population is very young with roughly 24% being 15–19 years old [25]. In the Bankim HD, we also observed a population that is strongly skewed towards young individuals and we found that living conditions and access to clean drinking water are very poor. Given the basic health infrastructure, these factors pose big challenges when addressing any health care related issues [26], [27].

Although Cameroon has achieved nationwide leprosy elimination as defined by the WHO (<1 case per 10'000 inhabitants) [28], our data showed that leprosy remains endemic in the Bankim HD. The substantial proportion of leprosy patients that had previously abandoned treatment further demonstrated that the oral treatment regimen requires better patient monitoring to achieve good compliance. In 2010 Cameroon reported 800 cases of yaws [29] and our survey confirmed that the Bankim HD is a yaws focus. Studies on the use of oral antibiotics have again raised hope for the eradication of yaws [30]–[32]. However, until eradication is possible, the focus of leprosy and yaws care should be early detection, complete cure and prevention of disabilities. To achieve this, front line medical staff needs to be trained on clinical diagnosis and efficient case management.

Characteristics of BU and the remote areas where it occurs have been suggested to necessitate active case searches for early case detection [33], [34]. Indeed, house-by-house surveys have helped to elucidate BU epidemiology in Ghana and Ivory Coast [35], [36]. In Cameroon a study around the Nyong River, identified 135 PCR confirmed cases of BU [6]. In the survey described here, the number of RT-PCR confirmed BU cases identified was smaller than expected. However, the lag of new cases during the first months after the survey indicated that the survey identified the cases present at that time. It cannot be excluded however, that a proportion of BU patients seeks to avoid contact with the formal health system. By continuous case detection, also accounting for the trust needed for cases to come forward [37], we identified 157 clinically diagnosed cases of BU (from April 2010 to June 2012). To increase validity of the findings [38], [39], our analysis focused on the 88 (56.1%) RT-PCR confirmed cases among them. False negative RT-PCR results are unlikely since we nalysed multiple samples from each patient (data not shown). Although accurate BU clinical diagnosis is possible [40], misdiagnosis rates of up to 40% have been reported emphasizing the pressing need for a point-of-care rapid diagnostic test [5], [38], [39], [41]–[43].

Based on the number of BU cases in each of the HA in the Bankim HD and their respective populations, the BR HA was determined to have the highest cumulative incidence rate of BU in the Bankim HD. Furthermore, by detailed mapping of cases and through the use of a geographic information system (GIS), we identified hot-spots of BU transmission along the Mbam River. With only few cases living in the immediate proximity of only the Mapé Dam reservoir, our data does not support the suspected direct importance of this man-made lake. This does not exclude that environmental changes associated with the damming of the Mapé River may have had a more indirect effect on the spread of BU in the wider area. Whether the relatively large proportion of patients living in the town of Bankim (11/79 GPS mapped cases), contracted BU there, remains to be investigated. By also mapping cases from outside of the Bankim HD, we found that the local BU endemic area is larger than previously described [7]. Indeed it is possible that, because of the differences in case finding strategy inside and outside of the Bankim HD, our findings from outside the HD under represent the true degree of BU endemicity in the areas surrounding the Bankim HD. Further studies are therefore needed to investigate BU endemicity in the entire Mapé basin in more detail. Ongoing environmental and social science research at the identified hot-spots of disease is aiming to further elucidate the mode of transmission of BU.

BU affects individuals of all ages [15], [34] but in the African endemic regions most patients are children [9], [44]. However, when adjusting for the population age distribution, studies in Benin [45] and in Australia [46] showed that 75 to 79 year olds or the ≥74 olds, respectively, have the highest risk of contracting BU. Our data similarly showed that the age adjusted risk of BU is as high in the >50 year olds as in children, a trend possibly associated with immunosenescence, the gradual deterioration of the immune system associated with natural age advancement [46], [47]. It is interesting to note that cases among very young (<5) children, which make up an even larger part of society than the 5–10 year olds, are relatively rare. This may indicate that compared to older children the very young children are less exposed to risk factors due to a smaller movement radius away from the house [10]. In the exposed individuals, host factors are likely to contribute to the degree of susceptibility [48]; seroepidemiological studies indicate that only a small proportion of exposed individuals develop clinical disease [49], [50].

Detection of *M. ulcerans* DNA-positive mosquitoes in an Australian BU focus [46] as well as identification of the failure to wear protective clothing as a risk factor and of the use of mosquito repellent as a protective factor for BU [8], support the hypothesis that insects are involved in *M. ulcerans* transmission [10]. Most biting arthropods selectively feed at specifics sites based on visual, physical or chemical cues such as distance of the ground, breath and skin temperature of the bait [51]–[55]. The resulting feeding patterns are often focused either on the feet and ankles or the head of the human subject [52]. Interestingly for vector transmitted parasitic diseases with local manifestations such as cutaneous leishmaniasis and filariasis, it has been found that the lesion distribution correlates with the biting sites of the responsible vectors [56], [57]. BU lesions occur mostly on the lower limbs [15], [45], [58]–[60] and in adults, a focus on joints, specifically the elbows and ankles, has been reported [15], [58]. Studies on the distribution of lesions also show that they are usually equally distributed between the left and right side of the body and compared to adults, children tend to have more lesions on the trunk [45], [60]. Using GIS methodology we observed in this study that lesions cluster at specific locations on the limbs. We found that, particularly in adults, lesions occur mostly at locations where the skin is not commonly protected with clothing. As previously described, in females, which are more likely to cover their upper body with clothing, we found that there are less lesions on the trunk. In rural African villages children may often have their upper body exposed explaining the more dispersed distribution of their lesions.

Detection of *M. ulcerans* DNA in the environment [10] and identification of poor wound care and failure to wear protective clothing as risk factors for BU [8] have led investigators to speculate that transmission may alternatively occur by skin trauma and direct contact with *M. ulcerans* contaminated environment [10]. A study in Canadian children found that children 9 months and older have on average >3.5 recent skin injuries [61]. In 5 to 17 year olds injuries most often occur where the bones are close to the skin, i.e. at shins, knees, elbows and forearms. Injuries on the head were most common in children less than 5 years of age and lesions on genitals were rare in all ages [61]. While this study may have, due to differences in dress code and activities, limited relevance for Cameroonian children, it is remarkable that both BU lesions on the head in our cohort occurred in patients under the age of 5 [61]. While BU lesion distribution does not seem to correlate closely with the published distribution of insect bites, inoculation of skin injuries by a contaminated environmental source should lead, for example, to more lesions on the knees. Based on these data, the option of multiple modes of transmission should be considered.

## Supporting Information

Checklist S1 **STROBE checklist.**
(PDF)

Dataset S1 **Shape file for the analysis of lesion distribution.** The compressed folder contains the files of the body_FB shapefile which provides the outline of the front and the back of the human body which was used for the “mapping” and analysis of the lesion distribution.
(ZIP)

Figure S1 **Disease category by health district.** Most of the RT-PCR confirmed cases that were identified in the Bankim area originated from within the Bankim HD (n = 62). However, patients from all of the surrounding HD also came to Bankim for BU treatment (Malantouen: 17; Nwa: 3; Yoko: 3; Mayo Drale: 1). The number of cases that occurred in each of the HD are classified by disease severity (red: category 3, orange: category 2, yellow: category 1). Two RT-PCR confirmed cases (both category 3, both from outside of the Bankim HD) could not be displayed because the location where the patient first showed symptoms of BU could not be conclusively determined.
(TIF)

Table S1 **Lesion distribution by body parts.**
(DOC)

Table S2 **Lesion on joints.**
(DOC)
