# Supplementary material for: Geographic Distribution, Age Pattern and Sites of Lesions in a Cohort of Buruli Ulcer Patients from the Mapé Basin of Cameroon
Source: PLoS Negl Trop Dis. 2013 Jun 13;7(6):e2252. doi: 10.1371/journal.pntd.0002252 (PMC3681622; doi:10.1371/journal.pntd.0002252)
Supplement: Checklist S1 — STROBE checklist. (PDF) [file pntd.0002252.s001.pdf]

STROBE Statement—checklist of items that should be included in reports of observational studies

	Item No	Recommendation
**Title and abstract**	1	(*a*) Indicate the study's design with a commonly used term in the title or the abstract<br>Title: "Geographic distribution, age pattern and sites of lesions in a Cohort of Buruli ulcer patients from the Mapé basin of Cameroon"
		(*b*) Provide in the abstract an informative and balanced summary of what was done and what was found<br>Abstract:<br>Buruli ulcer (BU), a neglected tropical disease of the skin, caused by Mycobacterium ulcerans, occurs most frequently in children in West Africa. Risk factors for BU include proximity to slow flowing water, poor wound care and not wearing protective clothing. Man-made alterations of the environment have been suggested to lead to increased BU incidence. M. ulcerans DNA has been detected in the environment, water bugs and recently also in mosquitoes. Despite these findings, the mode of transmission of BU remains poorly understood and both transmission by insects or direct inoculation from contaminated environment have been suggested. Here we investigated the BU epidemiology in the Mapé basin of Cameroon where the damming of the Mapé River since 1989 is believed to have increased the incidence of BU. Through a house-by-house survey in spring 2010, which also examined the local population for leprosy and yaws, and continues surveillance thereafter, we identified, till June 2012, altogether 88 RT-PCR positive cases of BU. We found that, the age adjusted cumulative incidence of BU was highest in young teenagers and in individuals above the age of 50 and that very young children (<5) were underrepresented among cases. BU lesions clustered around the ankles and at the back of the elbows. This pattern neither matches any of the published mosquito biting site patterns, nor the published distribution of small skin injuries in children, where lesions on the knees are much more frequent. The option of multiple modes of transmission should thus be considered. Analyzing the geographic distribution of cases in the Mapé Dam area revealed a closer association with the Mbam River than with the artificial lake.
**Introduction**		
Background/rationale	2	Explain the scientific background and rationale for the investigation being reported<br>See the INTRODUCTION.
Objectives	3	State specific objectives, including any prespecified hypotheses<br>The objectives of the present study were i) to conduct an exhaustive survey for BU, yaws and leprosy in the Bankim HD; ii) to continuously monitor the occurrence of BU in the Mapé Dam area; and iii) to examine the age distribution, geographic origin and distribution of lesions of the RT-PCR confirmed cases of BU to underpin future environmental and social science studies.
**Methods**		
Study design	4	Present key elements of study design early in the paper<br>In early 2010 (March 22 to April 19), we conducted an exhaustive cross-sectional house-by-house survey for BU, leprosy and yaws in the 88 villages of the Bankim HD (Figure 1).<br>Both BU cases identified in the survey and during the continuous case detection where included in the cohort of patients investigated here.

Setting	5	Describe the setting, locations, and relevant dates, including periods of recruitment, exposure, follow-up, and data collection<br>*Location:*<br>The study was conducted in the Mapé Dam region of Cameroon (Figure 1) at two different geographical scales. The initial phase of the study was conducted in the Bankim HD which consists of seven Health Areas (HA): Atta; Songkolong, Somié, Nyamboya, Bandam, Bankim Urban and Bankim Rural. For the later part of the study, bordering regions in the 4 HD surrounding the Bankim HD (Nwa HD, Malantouen HD, Mayo Darle HD, Yoke HD) were also included in the study area.<br>*Dates:*<br>In early 2010 (March 22 to April 19), we conducted an exhaustive cross-sectional house-by-house survey for BU, leprosy and yaws in the 88 villages of the Bankim HD (Figure 1). Following the survey, we continued to monitor the occurrence of all new cases of BU in the Bankim HD by community and HC based case referral and regular supervision until the end of June 2012.
Participants	6	(*a*) *Cohort study*—Give the eligibility criteria, and the sources and methods of selection of participants. Describe methods of follow-up<br>Both clinically confirmed BU cases identified in the survey and during the continuous case detection where included in the cohort of patients investigated here.<br>*Cross-sectional study*—Give the eligibility criteria, and the sources and methods of selection of participants<br>In early 2010 (March 22 to April 19), we conducted an exhaustive cross-sectional house-by-house survey for BU, leprosy and yaws in the 88 villages of the Bankim HD (Figure 1).
Variables	7	Clearly define all outcomes, exposures, predictors, potential confounders, and effect modifiers. Give diagnostic criteria, if applicable.<br>*Outcomes:*<br>To better describe BU epidemiology in the Mapé basin we set out to identify the exact geographic origin of all 88 laboratory confirmed cases in our cohort.<br>Age and gender distribution of cases<br>Localisation of BU lesions<br>*Diagnostic Criteria:*<br>Both clinically confirmed BU cases identified in the survey and during the continuous case detection where included in the cohort of patients investigated here.<br>To ensure the reliability of our conclusions we focused the remaining analysis only on the 88 RT-PCR confirmed BU cases.
Data sources/ measurement	8*	For each variable of interest, give sources of data and details of methods of assessment (measurement). Describe comparability of assessment methods if there is more than one group.<br>*Geographic origin:*<br>Based on information from the patients or their close relatives we were able to determine the exact origin of the BU cases in our cohort.<br>Age and gender distribution:<br>In addition to demographic and clinical information, the houses where the patients lived for at least a year before disease onset were mapped using a GPS device.<br>*Lesion localisation:*<br>Details of the location of the lesions on the patient's bodies were also collected and

		documented by photographs.
Bias	9	Describe any efforts to address potential sources of bias<br>To ensure the reliability of our conclusions we focused the remaining analysis only on the 88 RT-PCR confirmed BU cases.
Study size	10	Explain how the study size was arrived at<br>Our cohort study included all clinically diagnosed cases of BU which came for treatment in the Bankim HD during 28 months.
Quantitative variables	11	Explain how quantitative variables were handled in the analyses. If applicable, describe which groupings were chosen and why<br>Continuous variables were summarized as means and SD or medians and interquartile ranges and categorical ones as counts and percentages. The Fisher's exact or Chi-squared test were used to compare categorical characteristics between groups and Student t-tests or Mann-Whitney U-test in the case of continuous variables. Multiple comparisons were adjusted for using a Bonferroni correction.<br>Comparisons between laboratory confirmed and non-confirmed cases, cases and the general population, cases from inside and outside the Bankim HD and cases in adults and children were performed.
Statistical methods	12	(*a*) Describe all statistical methods, including those used to control for confounding<br>Continuous variables were summarized as means and SD or medians and interquartile ranges and categorical ones as counts and percentages. The Fisher's exact or Chi-squared test were used to compare categorical characteristics between groups and Student t-tests or Mann-Whitney U-test in the case of continuous variables. Multiple comparisons were adjusted for using a Bonferroni correction.
		(*b*) Describe any methods used to examine subgroups and interactions<br>Continuous variables were summarized as means and SD or medians and interquartile ranges and categorical ones as counts and percentages. The Fisher's exact or Chi-squared test were used to compare categorical characteristics between groups and Student t-tests or Mann-Whitney U-test in the case of continuous variables. Multiple comparisons were adjusted for using a Bonferroni correction.
		(*c*) Explain how missing data were addressed<br>For cross-sectional survey, only few study subjects had missing values and for the cohort study all necessary data could be collected. Some of the geographic data on the origin of the patients could not be obtained; patients for who this is the case are mentioned in the paper and approximations are described in as much detail as possible.
		(*d*) *Cohort study*—If applicable, explain how loss to follow-up was addressed<br>*Case-control study*—If applicable, explain how matching of cases and controls was addressed<br>NA.<br>*Cross-sectional study*—If applicable, describe analytical methods taking account of sampling strategy<br>In early 2010 (March 22 to April 19), we conducted an exhaustive cross-sectional house-by-house survey for BU, leprosy and yaws in the 88 villages of the Bankim HD (Figure 1).
		(*e*) Describe any sensitivity analyses<br>NA

Continued on next page

**Results**		
Participants	13*	(a) Report numbers of individuals at each stage of study—eg numbers potentially eligible, examined for eligibility, confirmed eligible, included in the study, completing follow-up, and analysed<br>In the course of the survey, a total of 48 962 individuals in 9 344 households (Figure 1) were interviewed.<br>Overall, our study identified 157 clinically confirmed cases of BU of which 88 (56%) could be confirmed by RT-PCR. These cases were analyzed in detail.
		(b) Give reasons for non-participation at each stage<br>The population of one village (approximately 550 people) refused to participate in the study.
		(c) Consider use of a flow diagram<br>NA
Descriptive data	14*	(a) Give characteristics of study participants (eg demographic, clinical, social) and information on exposures and potential confounders<br>Spatial distribution of BU cases in the Mapé basin<br>Age and gender distribution of cases<br>Localisation of BU lesions
		(b) Indicate number of participants with missing data for each variable of interest<br>*Spatial distribution of BU cases in the Mapé basin:*<br>Based on information from the patients or their close relatives we were able to determine the HD of origin for 86 (98%) of the cases (Figure S1). For the 62 cases that originated from within the Bankim HD we were also able to determine their HA of origin. Finally, for more detailed spatial analysis, the exact domiciles of 79 (89.8%) of the confirmed BU cases were mapped (Figure 3B).<br>*Age and gender distribution of cases:*<br>Information from all patients.<br>*Localisation of BU lesions:*<br>Information from all patients.
		(c) *Cohort study*—Summarise follow-up time (eg, average and total amount)<br>NA
Outcome data	15*	*Cohort study*—Report numbers of outcome events or summary measures over time<br>NA
		Case-control study—Report numbers in each exposure category, or summary measures of exposure<br>NA
		Cross-sectional study—Report numbers of outcome events or summary measures<br>Assessing demographics and living conditions in the Bankim HD (Table 1), we found that the local population is very young with an average age of 19.3±17.0 (median = 14.0, interquartile range = 6.0 to 28.0), that 51.4% of the population are women and that overall, 61.2% of the population have attended school at some point in their lives. We further observed that Christianity is the most common (64.9%) religion and that, apart from the young members of society which are either students (32.2%) or children (23.5%), the most common professions in the district are farming (16.9%) and household work (17.4%). In terms of living conditions we found that there are on average 5.2 individuals living in each household and 26.8% of the households have a mosquito net. Further, our data showed that only 38.3% of the population have access to clean drinking water that comes at least from a fortified well and that the roofs and floors of the local houses are often very poorly constructed.

Main results	16	(*a*) Give unadjusted estimates and, if applicable, confounder-adjusted estimates and their precision (eg, 95% confidence interval). Make clear which confounders were adjusted for and why they were included<br>NA
		(*b*) Report category boundaries when continuous variables were categorized<br>See Figure 4.
		(*c*) If relevant, consider translating estimates of relative risk into absolute risk for a meaningful time period<br>NA
Other analyses	17	Report other analyses done—eg analyses of subgroups and interactions, and sensitivity analyses<br>For the analysis of the lesion distribution, males and females and children and adults compared.
**Discussion**		
Key results	18	Summarise key results with reference to study objectives<br>In the course of the survey, a total of 48 962 individuals in 9 344 households (Figure 1) were interviewed. In the survey, we identified 32 cases of leprosy, 29 cases of yaws and 25 cases of BU based on clinical symptoms. Of the BU cases, 23% (6 cases) could be re-confirmed by RT-PCR.<br>In the five months after the survey (April 2010 to August 2010), only two new RT-PCR reconfirmed BU cases were identified (Figure 2). Following this lag, between September 2010 and June 2012 (22 months) there was a steady flow of about 2.5 new RT-PCR confirmed BU cases per month from the Bankim HD. During this period, RT-PCR confirmed BU patients from the surrounding HDs (about 1.2 per month) also reported to BU treatment facilities in the Bankim HD. Overall, our study identified 157 clinically confirmed cases of BU of which 88 (56%) could be confirmed by RT-PCR.<br>As shown in Figure 4B, we observed a low age adjusted cumulative incidence rate of BU in individuals aged below four years. The rate then peaked in children aged between four and < 14 years of age, with the 12 to <14 year olds particularly affected (34.4 cases per 10'000 inhabitants). Interestingly, the age adjusted cumulative incidence rate peaks again in the over 50 year olds (27.0 cases per 10'000 inhabitants; Figure 4B). This BU density map shows that most of the cases occur in the southern part of the Bankim HD, particularly along the Mbam River and in the area between the Mapé Dam reservoir and that river. Interestingly, most of the lesions (52.3%) occurred in close proximity to joints with clusters around the ankles (19.2 %) and elbows (15.9 %; Figure 5A, 5B and Table S2).
Limitations	19	Discuss limitations of the study, taking into account sources of potential bias or imprecision. Discuss both direction and magnitude of any potential bias<br>Indeed it is possible that, because of the differences in case finding strategy inside and outside of the Bankim HD, our findings from outside the HD under represent the true degree of BU endemicity in the areas surrounding the Bankim HD. Further studies are therefore needed to investigate BU endemicity in the entire Mapé basin in more detail.
Interpretation	20	Give a cautious overall interpretation of results considering objectives, limitations, multiplicity of analyses, results from similar studies, and other relevant evidence<br>This may indicate that compared to older children the very young children are less exposed to risk factors due to a smaller movement radius away from the house [10]. Based on the lesion distribution data, the option of multiple modes of transmission should be considered.
Generalisability	21	Discuss the generalisability (external validity) of the study results<br>With only few cases living in the immediate proximity of only the Mapé Dam reservoir, our

		data does not support the suspected direct importance of this man-made lake. This does not exclude that environmental changes associated with the damming of the Mapé River may have had a more indirect effect on the spread of BU in the wider area.
**Other information**		
Funding	22	Give the source of funding and the role of the funders for the present study and, if applicable, for the original study on which the present article is based<br>The work was funded by the Medicor Foundation (http://www.medicor.li/). The funders had no role in study design, data collection and analysis, decision to publish, or preparation of the manuscript.

*Give information separately for cases and controls in case-control studies and, if applicable, for exposed and unexposed groups in cohort and cross-sectional studies.

Note: An Explanation and Elaboration article discusses each checklist item and gives methodological background and published examples of transparent reporting. The STROBE checklist is best used in conjunction with this article (freely available on the Web sites of PLoS Medicine at http://www.plosmedicine.org/, Annals of Internal Medicine at http://www.annals.org/, and Epidemiology at http://www.epidem.com/). Information on the STROBE Initiative is available at www.strobe-statement.org.
