# Supplementary figures and images for: Geographic Distribution, Age Pattern and Sites of Lesions in a Cohort of Buruli Ulcer Patients from the Mapé Basin of Cameroon
Source: PLoS Negl Trop Dis. 2013 Jun 13;7(6):e2252. doi: 10.1371/journal.pntd.0002252 (PMC3681622; doi:10.1371/journal.pntd.0002252)

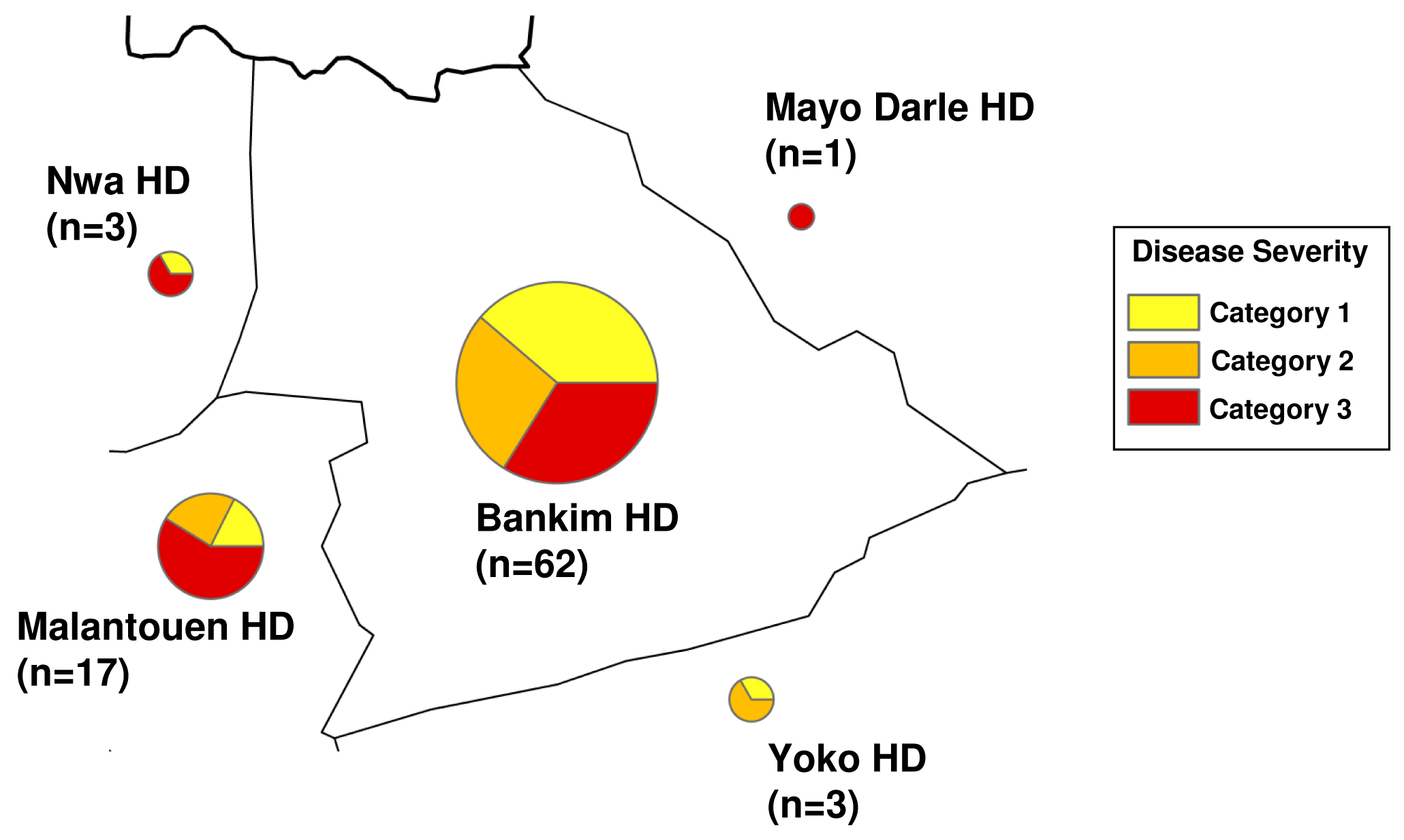

Supplement: Figure S1 — Disease category by health district. Most of the RT-PCR confirmed cases that were identified in the Bankim area originated from within the Bankim HD (n = 62). However, patients from all of the surrounding HD also came to Bankim for BU treatment (Malantouen: 17; Nwa: 3; Yoko: 3; Mayo Drale: 1). The number of cases that occurred in each of the HD are classified by disease severity (red: category 3, orange: category 2, yellow: category 1). Two RT-PCR confirmed cases (both category 3, both from outside of the Bankim HD) could not be displayed because the location where the patient first showed symptoms of BU could not be conclusively determined. (TIF) [file pntd.0002252.s003.tif]
