# Supplementary material for: Geographic Distribution, Age Pattern and Sites of Lesions in a Cohort of Buruli Ulcer Patients from the Mapé Basin of Cameroon
Source: PLoS Negl Trop Dis. 2013 Jun 13;7(6):e2252. doi: 10.1371/journal.pntd.0002252 (PMC3681622; doi:10.1371/journal.pntd.0002252)
Supplement: Table S1 — Lesion distribution by body parts. (DOC) [file pntd.0002252.s004.doc]

**Supplementary Table 1: Lesion Distribution by Body Parts.**

|  | **All** | | | **Children (< 15 years old)** | | | **Adults** | | |
| --- | --- | --- | --- | --- | --- | --- | --- | --- | --- |
| **Lesion location*** | **All (n=88)** | **Male (n=52)** | **Female (n=36)** | **All (n=52)** | **Male (n=34)** | **Female (n=18)** | **All (n=36)** | **Male (n=18)** | **Female (n=18)** |
| Head or neck | 2 (2.3) | 2 (3.8) | 0 (0) | 2 (3.8) | 2 (5.9) | 0 (0) | 0 (0) | 0 (0) | 0 (0) |
| Upper limbs | 27 (30.7) | 16 (30.8) | 11 (30.6) | 19 (36.5) | 14 (41.2) | 5 (27.8) | 8 (22.2) | 2 (11.1) | 6 (33.3) |
| Trunk | 10 (11.4) | 9 (17.3) | 1 (2.8) | 7 (13.5) | 7 (20.6) | 0 (0) | 3 (8.3) | 2 (11.1) | 1 (5.6) |
| Lower limbs | 49 (55.7) | 25 (48.1) | 24 (66.7) | 24 (46.2) | 11 (32.4) | 13 (72.2) | 25 (69.4) | 14 (77.8) | 11 (61.1) |

* number of patients with lesion at the given location and percentage in parenthesis
