# Supplementary material for: Geographic Distribution, Age Pattern and Sites of Lesions in a Cohort of Buruli Ulcer Patients from the Mapé Basin of Cameroon
Source: PLoS Negl Trop Dis. 2013 Jun 13;7(6):e2252. doi: 10.1371/journal.pntd.0002252 (PMC3681622; doi:10.1371/journal.pntd.0002252)
Supplement: Table S2 — Lesion on joints. (DOC) [file pntd.0002252.s005.doc]

**Supplementary Table 2: Lesion on Joints.**

|  | **All** | | | **Children (< 15 years old)** | | | **Adults** | | |
| --- | --- | --- | --- | --- | --- | --- | --- | --- | --- |
| **Lesion Location** * | **All (n=88)** | **Male (n=52)** | **Female (n=36)** | **All (n=52)** | **Male (n=34)** | **Female (n=18)** | **All (n=36)** | **Male (n=18)** | **Female (n=18)** |
| Ankle | 17 (19.3) | 10 (19.2) | 7 (19.4) | 4 (7.7) | 1 (2.9) | 3 (16.7) | 13 (36.1) | 9 (50.0) | 4 (22.2) |
| Elbow | 14 (15.9) | 9 (17.3) | 5 (13.9) | 10 (19.2) | 7 (20.6) | 3 (16.7) | 4 (11.1) | 2 (11.1) | 2 (11.1) |
| Hip | 3 (3.4) | 3 (5.8) | 0 (0) | 2 (3.8) | 2 (5.9) | 0 (0) | 1 (2.8) | 1 (5.6) | 0 (0) |
| Knee | 3 (3.4) | 3 (5.8) | 0 (0) | 3 (5.8) | 3 (8.8) | 0 (0) | 0 (0) | 0 (0) | 0 (0) |
| Shoulder | 2 (2.3) | 1 (1.9) | 1 (2.8) | 1 (1.9) | 1 (2.9) | 0 (0) | 1 (2.8) | 0 (0) | 1 (5.6) |
| Toe | 1 (1.1) | 0 (0) | 1 (2.8) | 0 (0) | 0 (0) | 0 (0) | 1 (2.8) | 0 (0) | 1 (5.6) |
| Wrist | 2 (2.3) | 2 (3.8) | 0 (0) | 2 (3.8) | 2 (5.9) | 0 (0) | 0 (0) | 0 (0) | 0 (0) |
| Not Joint | 46 (52.3) | 24 (46.2) | 22 (61.1) | 30 (57.7) | 18 (52.9) | 12 (66.7) | 16 (44.4) | 6 (33.3) | 10 (55.6) |

* number of patients with lesion at the given location and percentage in parenthesis
